# Role of Receptor Profiling for Personalized Therapy in a Patient with a Growth Hormone-Secreting Macroadenoma Resistant to First-Generation Somatostatin Analogues

**DOI:** 10.3390/jpm9040048

**Published:** 2019-11-15

**Authors:** Krystallenia I. Alexandraki, Eirini Papadimitriou, Vasiliki Mavroeidi, Georgios Kyriakopoulos, Antonios Xydakis, Theodoros G. Papaioannou, Denise Kolomodi, Gregory A. Kaltsas, Ashley B. Grossman

**Affiliations:** 1 Endocrine Unit, 1st Department of Propaedeutic Medicine, Laiko Hospital, Medical School, National and Kapodistrian University of Athens, 115 27 Athens, Greece; erin.papadimitriou@hotmail.com (E.P.); vasiliki.mavroeidi@gmail.com (V.M.); axydakis@hotmail.com (A.X.); denisekolomodi@hotmail.gr (D.K.); gkaltsas@endo.gr (G.A.K.); 2 Department of Pathology, Evangelismos Hospital, 115 27 Athens, Greece; geokyr11@hotmail.gr; 3 First Department of Cardiology, Medical School, National and Kapodistrian University of Athens, 115 27 Athens, Greece; thepap@med.uoa.gr; 4 Centre for Endocrinology, William Harvey Institute, Barts and the London School of Medicine, E1 2AT London, UK; ashley.grossman@ocdem.ox.ac.uk; 5 Oxford Centre for Diabetes, Endocrinology and Metabolism, University of Oxford, OX3 7LE Oxford, UK

**Keywords:** acromegaly, personalized treatment, somatostatin receptor, e-cadherin, resistant acromegaly, somatostatin analogue

## Abstract

Background: Acromegaly is almost always caused by a pituitary adenoma and is associated with high morbidity and mortality when uncontrolled. Trans-sphenoidal removal of the adenoma is the mainstay of therapy, but fails to control the disease in a significant number of patients who require further treatment. Somatostatin analogues (SSAs) as monotherapy or in combination with growth hormone (GH)-receptor antagonists and/or dopamine agonists are used either alone or in combination following surgical failure to achieve disease control. The use of specific biomarkers may help to individualize the therapeutic plan after surgical failure and direct towards a more personalized approach. Methods: We report a 41-year-old man with acromegaly and residual disease after repeated surgery that was resistant to first-generation SSAs. Results: Biochemical and tumor control were achieved following the administration of a second-generation SSA, pasireotide, combined with pegvisomant, both at maximal doses and along with cabergoline. Histology specimens showed a sparsely-granulated GH-immunostaining pituitary adenoma with intense positivity for somatostatin receptors 2 and 5 and low levels of E-cadherin. Conclusion: Personalized medical therapy guided by currently available biomarkers, such as immunohistochemically-characterized receptor profiling or adhesion molecules, resulted in controlled insulin-like growth factor-1 (IGF-1) and GH levels and symptom alleviation following the combination of three drug-classes.

## 1. Introduction

Acromegaly is a systemic disorder characterized by elevated and non-suppressible growth hormone (GH) and increased insulin-like growth factor-1 (IGF-1) levels, almost always caused by GH hypersecretion from a pituitary somatotroph adenoma [1]. Acromegaly has been associated with high mortality and morbidity, particularly when untreated or inadequately controlled [2,3,4,5], and its management includes surgery, medical therapy and radiotherapy. The therapeutic goals are the normalization of IGF-1 and lowering of mean serum GH below a threshold level, the control of tumor growth with preservation of normal pituitary function, along with the relief of symptoms and a decrease in associated comorbidities and/or mortality [6,7,8]. Normalizing IGF-1 levels has been suggested as the key therapeutic end-point, since this better reflects disease control [6,7,8].

Tumor resection is considered as first-line treatment of patients either with curative intent or when debulking is necessary for compressive symptoms to surrounding structures, or to increase sensitivity to medical therapy. Medical therapy is utilized as adjuvant treatment in patients with residual disease after surgery, or in patients who are not thought suitable for surgery [6,8]. Current therapies include three drug classes: first-generation (lanreotide and octreotide) or second-generation (pasireotide) somatostatin analogues (SSAs), dopamine agonists (mostly cabergoline), and GH-receptor antagonists (currently, only pegvisomant) [6,9]. Although a number of other compounds are in phase II studies they are currently not available for routine clinical practice. At present, first-generation SSAs [6] remain the preferred first-line pharmacological treatment. However, some 55% of patients do not obtain biochemical and/or tumor volume control [10,11,12,13], although this is usually only established after a ‘trial-and-error’ approach. Recently, it has been suggested that a more personalized approach to treatment may be established using a number of current and evolving biomarkers, such that we may be able to identify patients who could benefit from a particular drug or therapy, thus increasing the success rates of the available pharmacologic agents in acromegaly [6,9,13,14,15,16].

We describe here a patient where immunoreceptor profiling of somatostatin receptor type (SSTR) 2, SSTR5 [13,14,17] and cadherin E [18,19,20] were found to be useful to guide towards a more personalized therapeutic plan [3,13].

## 2. Case Presentation

**Figure 1:**
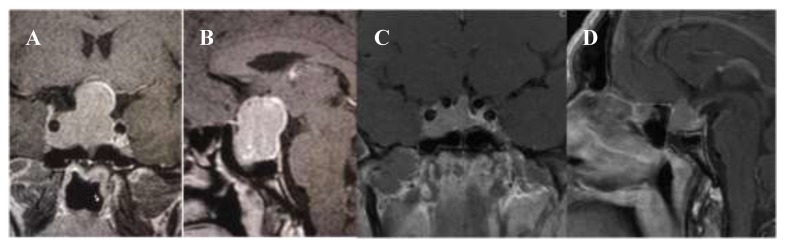
Magnetic resonance imaging showing macroadenoma (mass of 3.6 × 3.3 × 2.2 cm) in the sella turcica with right latero- and supra-sellar extension encasing the right internal carotid artery and compressing the optic chiasm: grade 4 according Knosp (**A**: coronal, **B**: sagittal plane); more recent MRI showing a decrease in the size of the macrodenoma, still extending towards the floor of the third ventricle and encasing the right cavernous sinus in the coronal plane (**C**); in the sagittal plane, a rather lobulated suprasella extension is shown but no evidence of stalk involvement (**D**). Initial laboratory testing confirmed an elevated serum IGF-1 of 988 (94-284) ng/mL, no suppression of GH after a 75 g glucose load (7 ng/mL), mild hyperprolactinemia and hypogonadism.

A 41-year-old man with acromegalic features [1] presented with bitemporal hemianopia. Magnetic resonance imaging (MRI) demonstrated a pituitary macroadenoma with right latero- and supra-sellar extension encasing the right internal carotid artery and compressing the optic chiasm [21] (Figure 1).

Lanreotide Autogel at a maximal dose of 120 mg every 4 weeks was initiated, in order to complete at least 3 months of treatment in an attempt to reduce the tumor bulk. However, because of emotional distress the patient decided to undergo trans-sphenoidal surgery (TSS), which proceeded uneventfully. Histology revealed a sparsely-granulated GH-immunostaining adenoma with expression of prolactin in scattered cells, and a Ki-67 labelling index (LI) of 1.2% and p53 expression < 5%. His vision normalized and symptoms of his acromegaly improved. Central hypothyroidism was confirmed and treated with thyroxine replacement.

**Figure 2:**
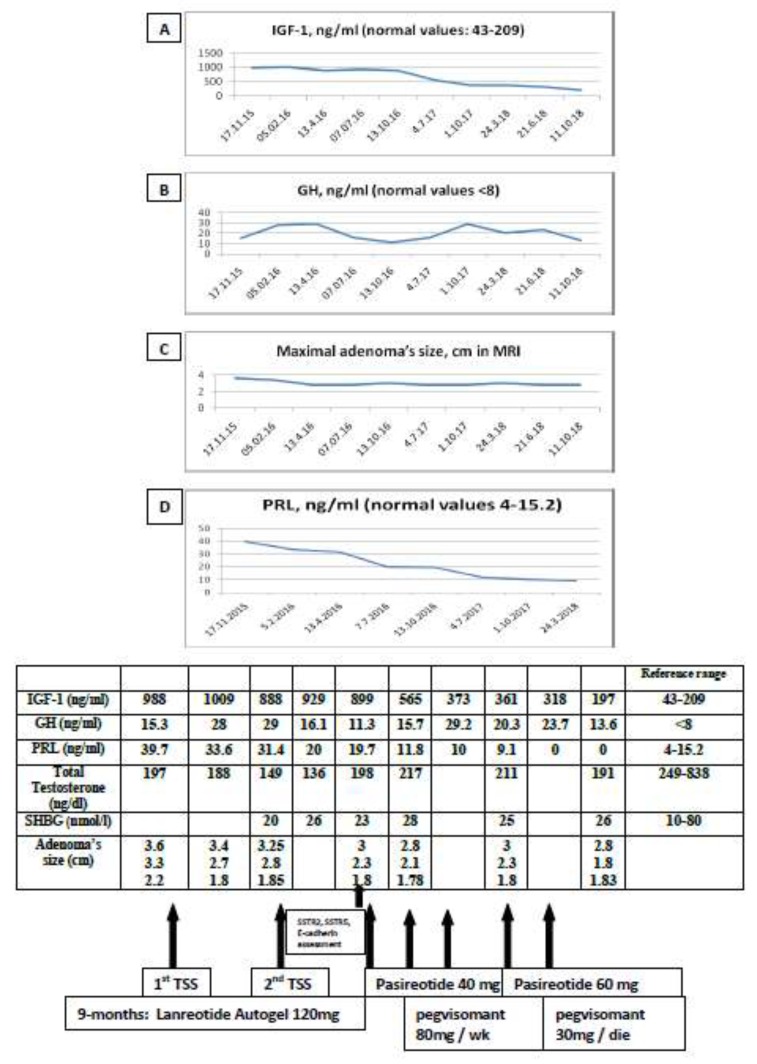
Pharmacological effects on serum IGF-1 (**A**), growth hormone (GH) (**B**), prolactin levels (**C**), and adenoma size (**D**) with different therapeutic modalities.

One month after surgery, the patient demonstrated a lack of biochemical control (Figure 2). Treatment with lanreotide Autogel was re-initiated; a repeat MRI showed slight debulking of the pituitary macroadenoma (Figure 2).

**Figure 3:**
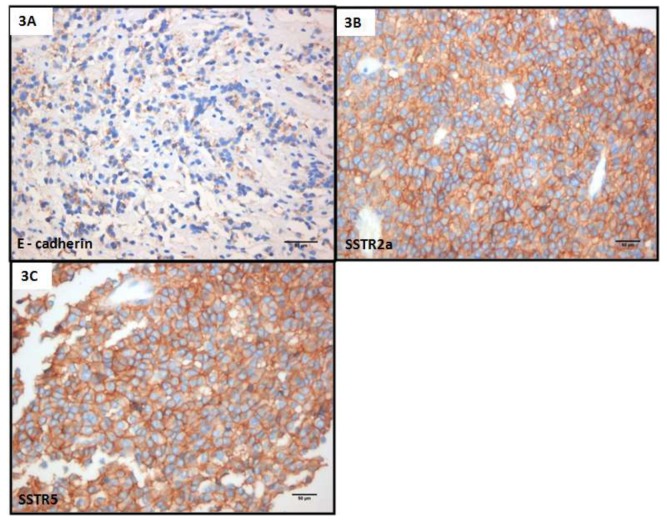
(**A**) Focally weak cytoplasmic expression of E-cadherin. (**B**,**C**) Circumferential membranous expression of SSTR2 and SSTR5, respectively, in more than 50% of the tumor cells, Volante score.

Five months later, he underwent a second TSS without complications. The histopathology reported widespread and intense membrane expression of SSTR2a, plus > 95% positive staining for SSTR5 and weak focal cytoplasmic expression of the adhesion molecule E-cadherin [18] (Figure 3). Three months later, a pituitary MRI showed an overall tumor volume reduction of 50%, with the optic chiasm free of tumor, but still without biochemical control. Since the patient was considered as resistant to first-generation SSAs, lanreotide Autogel was replaced with pasireotide as a long-acting releasing formulation (LAR) 40 mg every 4 weeks. Six months later, serum IGF-1 and GH decreased by 37% and 2.5% respectively, while no change was observed in the size of the adenoma. The patient had developed mild diabetes which was treated with metformin 1700 mg a day; pegvisomant 20 mg subcutaneous (SC) was also added four times a week. Two months later, a 34% decrease in serum IGF-1 levels was observed, but the level was still above the normal range and so the dose of pasireotide was increased to 60 mg a month. The patient reported clinical improvement but again without full biochemical control or further reduction of tumor size on MRI (Figure 1). Cabergoline 0.5 mg twice weekly was added and, after three months on combination treatment, serum IGF-1 decreased by a further by 12%. The dose of pegvisomant was subsequently increased to the maximal dose of 30 mg once daily: three months later, biochemical control was finally achieved, some 35 months since the initial presentation (Figure 2 and Figure 3). Significantly, during each step of his therapeutic management and because of the resistance to treatment, radiosurgery was offered to the patient, but he refused this treatment because of the possible late complication of radiation therapy of pituitary hormone insufficiency [6,8].

## 3. Discussion

The multidimensional use of combined treatment with pasireotide, pegvisomant, and cabergoline is highlighted in the case of our patient with acromegaly who was initially resistant to first-generation SSAs but, associated with the use of existing biomarkers, led to a more personalized treatment plan and ultimately biochemical control of the disease.

It has recently been acknowledged in consensus guidelines that the absence of response to a first-generation SSA may lead to the use of a second-generation SSA, particularly in a patient with only mild carbohydrate abnormalities and in the presence of a significant tumor volume, rendering pegvisomant a less favorable choice [6,22,23]. Pasireotide was selected because of the increased expression of SSTR5 in the tumor tissue. The superior effect of pasireotide LAR over octreotide LAR at suppressing IGF-1 but displaying a similar effect on GH inhibition has previously been demonstrated. This is probably attributable to the higher affinity and functional activity of pasireotide at hepatic SSTR1, 3, or 5 [17,24]. However, suppression of insulin release by pasireotide and possible antagonism of the action of GH on hepatic IGF-1 may also contribute to the lowering of IGF1 levels [25], underlining a complex mechanism of SSA action not explained by a direct inhibition of GH release alone [26].

A number of factors might predict a poor response to lanreotide Autogel in the present patient which could have been considered before treatment, thus obviating the need and additional cost of a non-efficacious treatment. Young age at presentation, male gender, high baseline hormonal levels, high signal intensity on T2-weighted MRI, and the histopathology of a sparsely-granulated pattern [13,27] with intense expression of SSTR5 and low expression of E-cadherin [13,14,18,28], are all markers pointing to an expected poor response to a predominant SSTR2 agonist such as lanreotide. Pasireotide is a second-generation SSA with higher affinity for SSTR5 (besides SSTR1,-2,-3) [24,28]. The PAOLA study has shown that a six-month treatment with pasireotide 40 mg produced a mean fall of 23% in GH and 28% in IGF1, with respective figures of 51% and 39% on 60 mg monthly, in patients resistant to first-generation SSAs [9,28]. The ACRONIS study, an observational study of patients resistant to first-generation SSAs, showed significant hormonal control after 6-months of treatment with any dose of pasireotide [29]. In the PAPE study, a well-controlled study of patients with acromegaly on a LAR first-generation SSA and pegvisomant combination, changing to pasireotide LAR with or without pegvisomant led to a > 50% reduction in the dose of pegvisomant necessary to achieve similar disease-control [30]. Thus, the addition of pegvisomant and the increase in pasireotide dosage represents a valid strategy to optimize biochemical control. However, it has also to be accepted that this combination may increase the possibility of complications such as diabetes, and the drug is of high cost. The combination of pasireotide LAR, pegvisomant, and cabergoline was also used in an Italian cohort of patients with aggressive resistant acromegaly [31], while a meta-analysis suggested that the addition of cabergoline in patients resistant to first-generation SSAs resulted in normalization of IGF-1 in around 50% of cases, even in patients with normoprolactinemia [32]. However, in this specific case its efficacy and value remain uncertain, and it is usually most useful where levels of IGF1 are only mildly elevated [6]. However, its low cost and its ease of administration were the main reasons that we included this agent in our therapeutic scheme.

A major disadvantage of first-line pasireotide use is mainly due to its hyperglycemic effect (ACCESS/PAPE) [30,33,34]. However, a recent meta-analysis demonstrated that pegvisomant dosage may be adjusted accordingly to counteract the negative impact of pasireotide [30,35]. This phenomenon is considered to be related to decreased secretion of insulin and incretins, without concomitant changes in hepatic or peripheral insulin sensitivity [34]. Since glucagon-producing *α*-cells only express SSTR2, whereas insulin-producing *β*-cells express both SSTR2 and 5, pasireotide selectively suppresses insulin secretion. In a one-week phase I study in healthy volunteers, vildagliptin and liraglutide proved more efficient in controlling hyperglycemia related to pasireotide compared to metformin, confirming the involvement of incretins [34]. Consequently, before initiating pasireotide therapy glucose metabolism should be assessed, and in patients with preexisting diabetes anti-diabetic treatment should be optimized.

**Figure 4:**
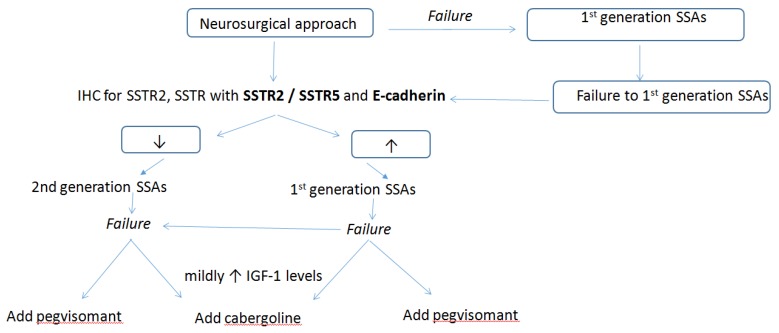
Suggested algorithm of the use of biomarkers in clinical practice. insulin-like growth factor-1 (IGF-1); IHC: immunohistochemistry; SSAs: somatostatin analogues; SSTR: somatostatin receptor.

Immunohistochemistry of somatotroph tumors has been problematic, with heterogeneity in tumoral SSTR expression in different studies, variability of techniques to detect SSTR status, and the pre-operative treatment of tumors, all contributing to highly variable results [13,14]. In cases where both SSTR2/5 are intensively positive on IHC, an additional factor such as E-cadherin may guide further treatment [13,27]. It has been suggested that additional biomarkers could be used to predict the biological behavior of the tumor and its response to treatment. The *Molecular Registry of Pituitary Adenomas* (REMAH study) is a multicenter, interdisciplinary network founded on a shared database that provides a translational approach for the personalized management of pituitary adenomas by combining clinical, pathological, and molecular information [13,36]. Preliminary results from the study have confirmed the concept that GH-secreting adenomas are heterogeneous tumors with a highly variable molecular expression of genes associated with SSAs, and further suggested that E-cadherin is the best molecular discriminator of a therapeutic response to SSAs [37]. Cadherins are transmembrane glycoproteins responsible for a calcium-dependent process of cell-cell adhesion. A lack of cadherin expression may be indicative of cellular dedifferentiation and the metastatic potential of tumors [19]. Reduced E-cadherin expression has been suggested to be associated with a dedifferentiated phenotype in somatotroph pituitary adenomas [38]. Recent studies have confirmed that E-cadherin-negative somatotroph pituitary adenomas were mostly sparsely-granulated, and generally larger. Regarding responses to treatment with SSAs, at six months the median IGF-1 reduction for adenomas negative for E-cadherin was 8.9% compared to 49.8% in adenomas positive for E-cadherin [18]. Currently, there are no other biomarkers which have been validated in clinical practice [13,14,18,36,37,39,40]. We therefore present a suggested algorithm for the use of biomarkers in clinical practice (Figure 4).

Information from many different national registries showed that adequate control of acromegaly remains a major problem in clinical practice, ranging from 35% to 73% [15,16,41,42]. Thus, a cost-effective panel of biomarkers may be useful in predicting responses to the currently approved medical treatments, and thereby optimizing effective management. Another way to reduce the rate of uncontrolled patients is the introduction of new agents that can better control the disease or improve compliance. New pharmaceutical agents related to SSAs have been introduced such as the oral octreotide capsules [43] or octreotide implants [44]. Furthermore, pharmacological agents unrelated to SSAs such ATL1103, a second-generation antisense oligomer targeting the GH receptor, are currently under investigation [45] (Table 1).

## 4. Conclusions

In conclusion, combined treatment with pasireotide, pegvisomant, and cabergoline has been shown to be safe and effective in achieving biochemical control and clinical improvement in a patient with acromegaly resistant to first-generation SSAs after repeated surgery. Receptor profiling, the use of biomarkers and the molecular characterization of the pituitary tumors, may guide an individualized therapeutic plan and thus limiting the cumulative GH exposure. New pharmacological agents are also under investigation to allow for better compliance and effectiveness.

## Figures and Tables

**Table 1:** New pharmaceutical agents currently used or in future use for the medical therapy of acromegaly.

Related to SSA Therapies		
Agents		
Octreotide LAR	▪ Affinity for SSTR2, slight for 5 ▪ Intramuscular (IM) Administration every 4 weeks	EMA/FDA approved
Lanreotide autogel	▪ Affinity for SSTR2, slight for 5 ▪ Deep subcutaneous (SC) Administration every 4–6 weeks	EMA/FDA approved
Pasireotide LAR	▪ affinity for SSTR1, 2, 3, 5 ▪ intramuscular (IM) Administration every 4–6 weeks	EMA/FDA approved
Octreotide capsules	20 mg per os daily = 0.1 mg octreotide SC three times daily (Avoid food and PPI)	Under completion of phase III trials. FDA not approved.
Octreotide implant	Stable dosing more than three months	Under phase II trials
Factor CAM2029	▪ Octreotide binding in a liquid mould with affinity for SSTR2, 5 ▪ 20 mg sc/≥4 weeks, 10 mg sc/2 weeks	Phase II trials completed FDA approved to begin phase III trials
Factor PTR 3173 (Somatoprim)	▪ Somatostatin receptor ligand with high selectivity for GH suppression ▪ Affinity for SSTR2, 4, 5 im Administration every 4 weeks	Phase II trial: more selective and effective GH inhibition without effect on insulin secretion compared to octreotide; better response of sparsely granulated adenomas
**Unrelated to SSA Therapies**		
Pegvisomant	▪ GH receptor antagonist ▪ Daily SC administration	EMA/FDA approved
Factor ALT1103	▪ Oligonucleotide for the GH receptor ▪ SC administration	Under phase II trials
Dopastatin (BIM-23A760/BIM-065)	▪ D2R chimeric receptor binding to D2 and SSTR2, -5 ▪ More potent and without intermediate metabolites but its efficacy decreases over time due to metabolite	Under phase II trials
Τemozolomide	150 mg/m^2^ (5 days) every 28 days Chemotherapy agent	EMA/FDA approved for glioblastoma multiforme
Botulinum toxin molecule	Chimeric molecule that binds to cells expressing GHRH receptors to induce GH inhibition	No current trial
